# Atypical primary central nervous system lymphoma and glioblastoma: multiparametric differentiation based on non-enhancing volume, apparent diffusion coefficient, and arterial spin labeling

**DOI:** 10.1007/s00330-023-09681-2

**Published:** 2023-05-12

**Authors:** Xiaojun Yu, Weiping Hong, Minting Ye, Mingyao Lai, Changzheng Shi, Linzhen Li, Kunlin Ye, Jiali Xu, Ruyu Ai, Changguo Shan, Linbo Cai, Liangping Luo

**Affiliations:** 1 Medical Imaging Center, The First Affiliated Hospital of Jinan University, No. 613, Huangpu Road West, Tianhe District, Guangdong Province Guangzhou, 510630 China; 2 Department of Oncology, Guangdong Sanjiu Brain Hospital, Guangzhou, 510510 China

**Keywords:** Central nervous system, Lymphoma, Glioblastoma, Magnetic resonance imaging, Tumor volume

## Abstract

**Objectives:**

To evaluate the multiparametric diagnostic performance with non-enhancing tumor volume, apparent diffusion coefficient (ADC), and arterial spin labeling (ASL) to differentiate between atypical primary central nervous system lymphoma (PCNSL) and glioblastoma (GBM).

**Methods:**

One hundred and fifty-eight patients with pathologically confirmed typical PCNSL (*n* = 59), atypical PCNSL (hemorrhage, necrosis, or heterogeneous contrast enhancement, *n* = 29), and GBM (*n* = 70) were selected. Relative minimum ADC (rADC_min_), mean (rADC_mean_), maximum (rADC_max_), and rADC_max-min_ (rADC_dif_) were obtained by standardization of the contralateral white matter. Maximum cerebral blood flow (CBF_max_) was obtained according to the ASL-CBF map. The regions of interests (ROIs) were manually delineated on the inner side of the tumor to further generate a 3D-ROI and obtain the non-enhancing tumor (nET) volume. The area under the curve (AUC) was used to evaluate the diagnostic performance.

**Results:**

Atypical PCNSLs showed significantly lower rADC_max_, rADC_mean_, and rADC_dif_ than that of GBMs. GBMs showed significantly higher CBF_max_ and nET volume ratios than that of atypical PCNSLs. Combined three-variable models with rADC_mean_, CBF_max_, and nET volume ratio were superior to one- and two-variable models. The AUC of the three-variable model was 0.96, and the sensitivity and specificity were 90% and 96.55%, respectively.

**Conclusion:**

The combined evaluation of rADC_mean_, CBF_max_, and nET volume allowed for reliable differentiation between atypical PCNSL and GBM.

**Key Points:**

• *Atypical PCNSL is easily misdiagnosed as glioblastoma, which leads to unnecessary surgical resection.*

• *The nET volume, ADC, and ASL-derived parameter (CBF) were lower for atypical PCNSL than that for glioblastoma.*

• *The combination of multiple parameters performed well (AUC = 0.96) in the discrimination between atypical PCNSL and glioblastoma.*

**Supplementary Information:**

The online version contains supplementary material available at 10.1007/s00330-023-09681-2.

## Introduction

Primary central nervous system lymphoma (PCNSL) and glioblastoma (GBM) are the two most common primary malignant brain tumors [1]. They are handled differently; the treatment for GBM is wide surgical resection combined with temozolomide radiation therapy and chemotherapy [2], whereas that for PCNSL is high-dose methotrexate basic chemotherapy after stereotactic biopsy [3]. In most cases, magnetic resonance imaging (MRI) sequences can distinguish between the two tumors as PCNSL usually presents as a solitary, uniformly enhancing mass in immunocompetent patients [4], while GBM typically presents as a nonhomogeneous enhancement with obvious necrosis [5]. In patients without acquired immune deficiency syndrome (AIDS), the imaging features of PCNSL (including hemorrhage, necrosis, or heterogeneous contrast enhancement) are usually atypical as they nearly resemble GBM [6], making it difficult to differentiate between PCNSL and glioblastoma. Therefore, accurate differential diagnosis is the key to improving the therapeutic effects, avoiding unnecessary surgical resection, and protecting the nervous system function.

Previous studies used various advanced MRI quantitative techniques to distinguish PCNSL from GBM or atypical GBM [7–18], such as dynamic contrast-enhanced MRI, dynamic susceptibility-weighted contrast-enhanced perfusion-weighted imaging, diffusion-weighted imaging (DWI), and arterial spin labeling (ASL) to reflect the heterogeneity of tumor diffusion and perfusion. However, only a few studies focused on distinguishing atypical PCNSL and GBM. Suh et al [6] and Kang et al [19] introduced the intravoxel incoherent motion and diffusion radiomics, respectively, and the latter carries out multicenter external validation, with positive results. However, there is a gap in the quantitative study on using the volume of interest (VOI) (non-enhancing tumor, whole tumor) to distinguish between atypical PCNSL and GBM. The purpose of our study was to evaluate the feasibility and diagnostic performance of various MRI features such as non-enhancing tumor (nET) volume ratio, apparent diffusion coefficient (ADC), and ASL to differentiate atypical PCNSL from GBM in non-AIDS patients.

## Materials and methods

Our institutional review committee approved this retrospective study and waived the requirement of informed consent.

### Study participants

**Fig. 1:**
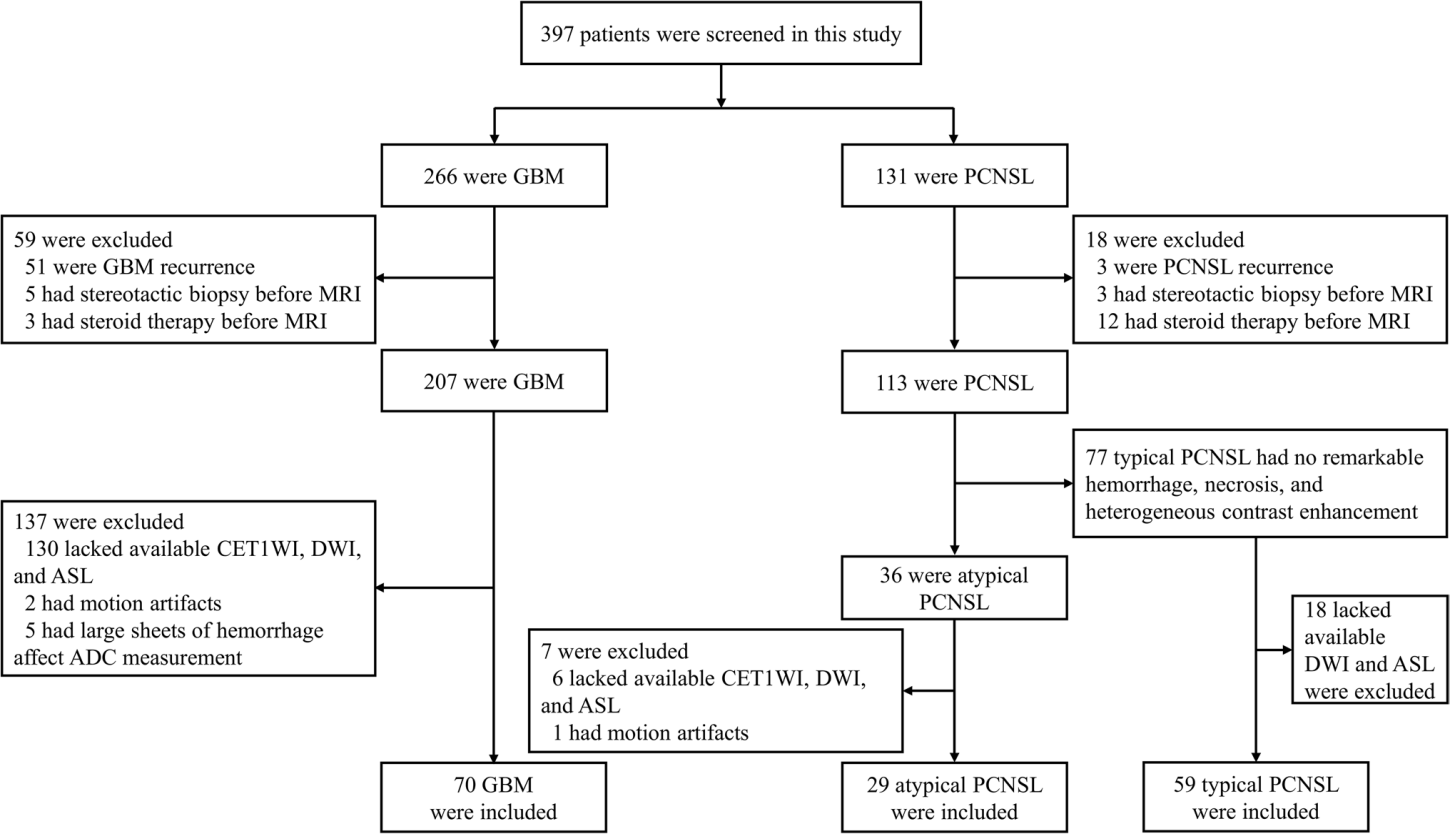
Flow diagram for the patient selection process. PCNSL, primary CNS lymphoma; GBM, glioblastoma; CET1WI, contrast-enhanced T1WI; DWI, diffusion-weighted imaging; ASL, arterial spin labeling

Between July 2017 and September 2021, 397 consecutive patients whose histopathological diagnoses were confirmed as GBM (*n* = 266) or PCNSL (*n* = 131) were identified. Among these, 239 patients were excluded due to multiple reasons (Fig. 1). The pretreatment MRI of each PCNSL patient was evaluated by two independent readers (with 4 and 15 years of radiology imaging experience, respectively) to determine the presence of atypical imaging features. Atypical imaging features include hemorrhage, necrosis, or heterogeneous contrast enhancement [6, 19]. When two independent readers judged that there were atypical imaging findings at the same time, we included the patients in the atypical PCNSL group. Finally, 158 non-AIDS patients (70 GBMs, 29 atypical PCNSLs, and 59 typical PCNSLs) were enrolled. Two observers (with 3 and 4 years of experience in radiology, respectively), who were blinded to the diagnosis, provided their diagnostic opinions on the 29 cases of atypical PCNSL. The diagnosis of PCNSL is based on the pathological examination. Atypical and typical PCNSLs are the results of this study reviewed by two readers. Among the 29 patients with atypical PCNSL, 11 (37.9%) underwent stereotactic biopsy, and 18 (62.1%) underwent subtotal or total resection. Among the 59 patients with typical PCNSL, 42 (71.2%) underwent stereotactic biopsy, and 17 (28.8%) underwent subtotal or total resection. Among the 70 patients with GBM, six (8.6%) underwent stereotactic biopsy, and 64 (91.4%) underwent subtotal or total resection.

### MRI parameters

MR images were acquired by using a 3.0-T (Signa HDxt; GE Healthcare) or 1.5-T (Achieva; Philips Medical Systems) MR scanner with an 8-channel head coil. Imaging sequences included T1WI, T2WI, T2-FLAIR, contrast-enhanced T1WI (CET1WI), DWI, and ASL-PWI. ASL-PWI imaging was performed using the GE Signa HDxt 3.0 T MRI machine. The following parameters were used for T1WI: TR, 488–1900 ms, TE, 15–24 ms; T2WI: TR, 4480–6000 ms, TE, 120 ms; T2-FLAIR: TR, 7780–9480 ms, TE 120–135 ms, FOV, 240 × 240 mm^2^, matrix, 256 × 256, slice thickness, 5.5 mm (with a gap of 1 mm), the number of excitations (NEX), 1; and DWI: TR 2262–6000 ms, TE 74.7–75 ms, matrix 256 × 256, slice thickness 5.5 mm; FOV, 240 × 240 mm, *b* = 0, and 800/1000 s/mm^2^.

3D-pCASL images were acquired with a spiral-fast spin-echo sequence. The MRI parameters were as follows: TR, 4599 ms; TE, 9.8 ms; slice thickness, 4 mm; number of excitations, 3; number of slices, 36; FOV, 240 × 240 mm; matrix, 512 × 512; post labeling delay time, 1525 ms; and scan time, 4 min 21 s.

### Image processing and analysis

**Fig. 2:**
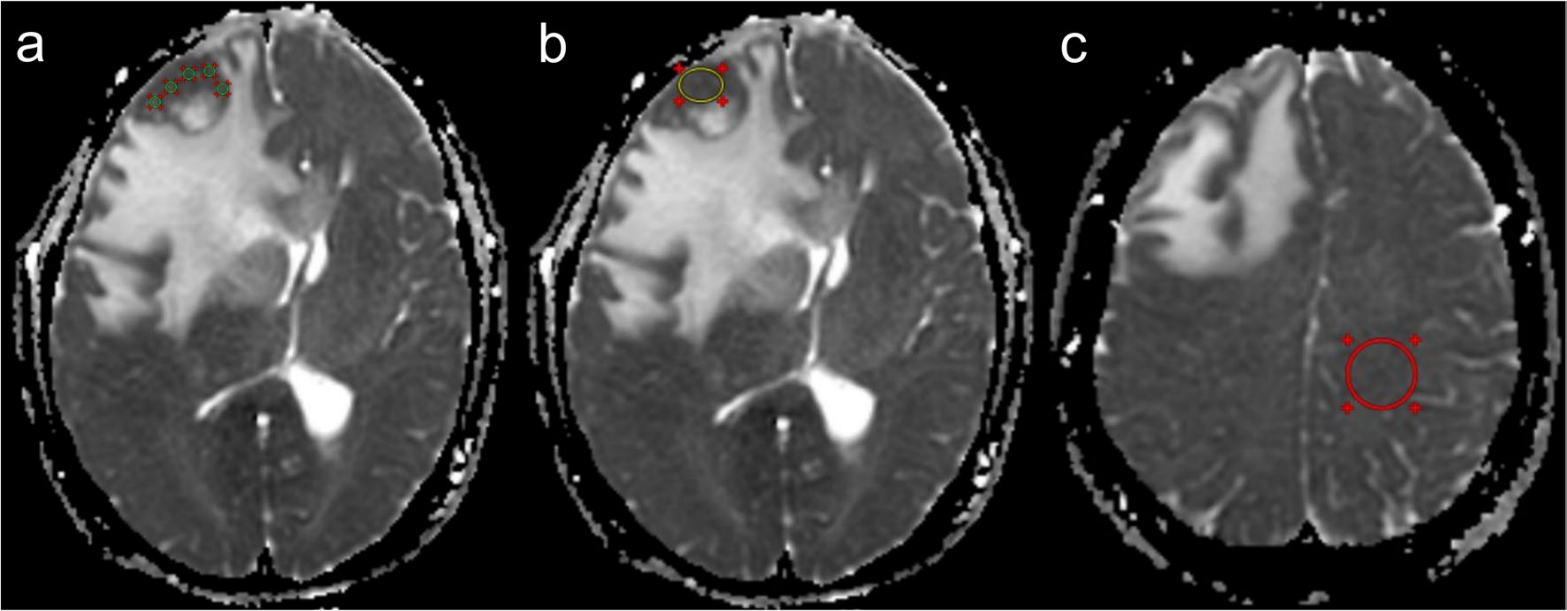
ADC measurements. **a** Minimum and maximum ADC (≥ 5 per patient; green). **b** Mean ADC (yellow). **c** Normal-appearing white matter ADC (red). ADC, apparent diffusion coefficient

All MR images were reviewed by two observers (with 3 and 4 years of radiology imaging experience, respectively) who were blinded to diagnosis. For quantitative ADC, CBF, nET, and wT volume measurements, both readers performed the region of interest (ROI) analysis. Multifocal tumors were measured in larger lesions. For each ROI, the average of the two observer’s measurements was used as the final value. The ADC measurement was performed using the off-line software RadiAnt DICOM Viewer (Medixant. RadiAnt DICOM Viewer [Software]. Version 2021.1. Jun 27, 2021. URL: https://www.radiantviewer.com) as shown in Fig. 2. ADC measurements included 158 patients (70 GBMs, 29 atypical PCNSLs, and 59 typical PCNSLs). Five or more circular ROIs (5–20 mm^2^) were placed on the solid component (multiple random larger layers) of the tumor. The lowest and maximum ADC values obtained by the placed ROI were regarded as ADC_min_ and ADC_max_, as reported in the study by Xing et al [20]. ADC_max-min_ was designated as ADC_dif_. We plotted a large ROI (ADC_mean_) to cover the largest tumor axial cross-section while avoiding areas of calcification, bleeding, and necrosis. A large ROI (ADC_NAWM_) was placed in the contralateral normal-appearing white matter, as reported in the study by John et al [21]. Finally, we normalized ADC_min_, ADC_max_, ADC_mean_, and ADC_dif_ to the contralateral normal-appearing white matter to obtain the relative ADC_min_, ADC_max_, ADC_mean_, and ADC_dif_ values (rADC_min_, rADC_max_, rADC_mean_, and rADC_dif_).

The AW4.6 workstation (GE Healthcare) was used to measure CBF. The CBF measurement data included 158 patients (70 GBMs, 29 atypical PCNSLs, and 59 typical PCNSLs). The CBF pseudo-color image was obtained by post-processing the original image. Subsequently, the CBF maps and T1WI images are fused manually. For each tumor, three circular ROIs (area > 30 mm^2^) were placed on the CBF maps with reference to the enhancing area on the contrast-enhanced T1WI images. From these, the maximum CBF ROI measurement was designated as CBF_max_.

### VOI measurement

**Fig. 3:**
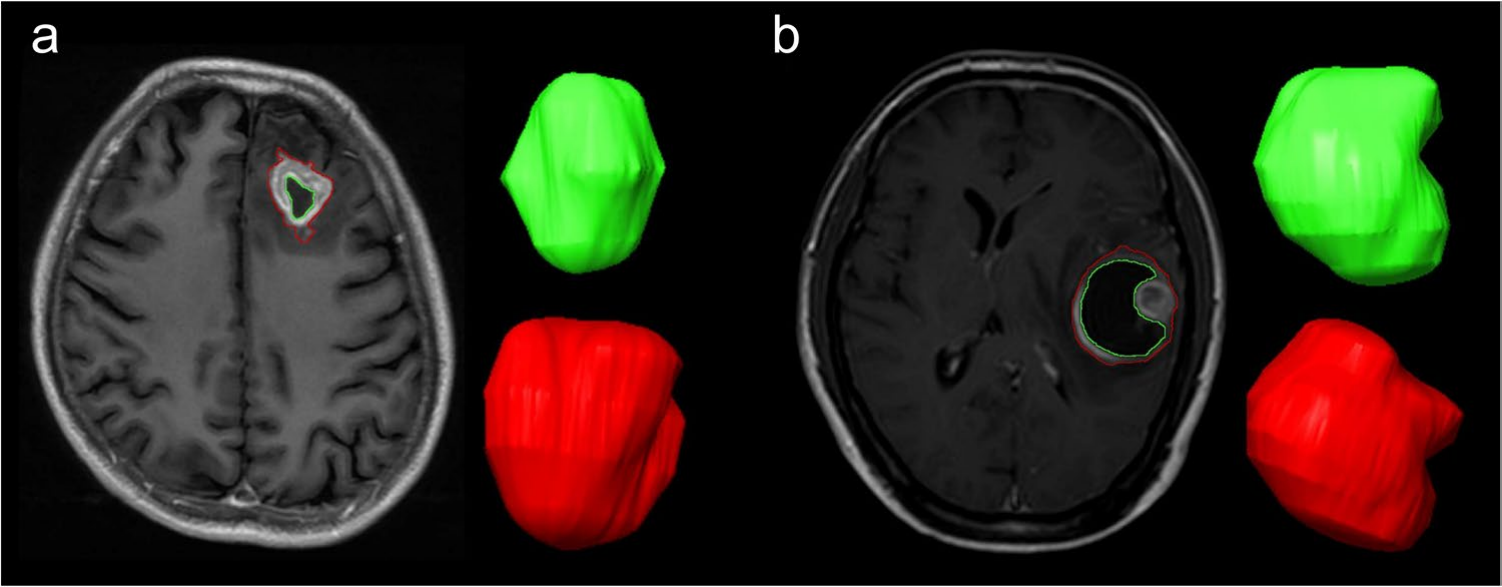
ROI measurement. Green represents nET volume and red represents wT volume. **a** Male, 38 years, PCNSL, nET volume ratio was 15.5%. **b** Male, 38 years, GBM, nET volume ratio was 58.2%. ROI, region of interest; nET, non-enhancing tumor; wT, whole tumor; PCNSL, primary CNS lymphoma; GBM, glioblastoma

The iplan RT Image 4.1 2 (BrainLAB) was used for VOI measurements. VOI measurements included 158 patients (70 GBMs, 29 atypical PCNSLs, and 59 typical PCNSLs). Post-contrast Axial CET1WIs were used to measure the whole tumor and non-enhancing area volume. The ROI was manually delineated layer by layer along the inner/outer margins of the visible tumor, as reported in the study by Wu et al [22] to further generate a 3D-ROI and obtain the volume of the nET, whole tumor (wT), and the ratio nET/wT, as shown in Fig. 3.

### Statistical analysis

All statistical analyses were performed using IBM SPSS Statistics for Windows, version 26.0 (IBM Corp), and MedCalc version 19.6.4 (MedCalc Software). *p* < 0.05 was considered statistically significant. The intraclass correlation coefficient (ICC) was used to evaluate the interobserver agreement of ADC values, ASL perfusion parameters, and tumor volume parameters. The Mann–Whitney U test or *t*-test was used to compare continuous variables (age and results of quantitative analysis) between PCNSL and GBM. The Mann–Whitney U test or *t*-test was used to compare the non-enhancing tumor volume ratio between atypical PCNSL and GBM. One-way ANOVA and the Kruskal–Wallis H test were used to compare continuous variables (age and results of quantitative analysis) between atypical PCNSL, typical PCNSL, and GBM. The chi-square test was used to compare the sex, tumor location, and the number of lesions between PCNSL and GBM, atypical PCNSL, typical PCNSL, and GBM. Bonferroni correction was used to correct for multiple tests and use the adjusted Significance value. Data following a normal distribution are expressed as mean ± standard deviation. Data that do not follow a normal distribution are expressed as median (first and third quartiles). For parameters demonstrating a significant difference between the two tumor types, an assessment of the feature’s ability to discriminate the two lesion types was undertaken using receiver operating characteristic (ROC) derived area under the curve (AUC) analysis. According to the maximum Youden index obtained from the ROC curve, the sensitivity and specificity were calculated through the optimal cut-off points.

Using the parameters with the highest AUC values, multiparameter logistic regression, including two or three of these imaging parameters, was generated to ascertain the best diagnostic model. ROC analysis was performed to evaluate the diagnostic performance of each model.

## Results

This study included 158 patients (70 GBMs, 29 atypical PCNSLs, and 59 typical PCNSLs). The PCNSLs and GBMs information and qualitative imaging features are summarized in Table 1. The atypical PCNSLs, typical PCNSLs, and GBMs information and qualitative imaging features are summarized in Table 2. There was no significant difference in sex, age, and qualitative imaging features between the three groups. In 11 patients with atypical PCNSL, both observers gave the diagnosis of GBM concurrently. Five patients with atypical PCNSL were misdiagnosed as GBM by one of the two observers. In 13 patients with atypical PCNSL, both observers gave the diagnosis of PCNSL concurrently. Among the 29 patients with atypical PCNSL, 24 received intraoperative pathological examination, of which 22 were diagnosed as lymphoma, 2 were diagnosed as round cell malignant tumors, and 5 did not receive intraoperative pathological examination. More typical PCNSL intraoperative pathological details are shown in Supplementary Table 1.

**Table 1:** Comparison of patient information, qualitative imaging features, and quantitative parameters (ADC, ASL-derived, and volume) between PCNSL and GBM

	PCNSL (*n* = 88)	GBM (*n* = 70)	*p* value
Age (years)	54.50 (45.25–64.00)	52.00 (43.00–58.00)	0.120
Sex			0.993
Male	54 (61.4%)	43 (61.4%)	
Female	34 (38.6%)	27 (38.6%)	
Location			0.094
Deep brain	52 (59.1%)	32 (45.7%)	
Not deep brain	36 (40.9%)	38 (54.3%)	
Number of lesions			0.098
Single	49 (55.7%)	48 (68.6%)	
Multiple	39 (44.3%)	22 (31.4%)	
rADC_max_	1.26 (1.18–1.35)	1.53 (1.39–1.59)	< 0.001^***^
rADC_mean_	0.92 ± 0.13	1.18 ± 0.17	< 0.001^***^
rADC_min_	0.70 (0.58–0.78)	0.72 (0.66–0.84)	0.010^*^
rADC_dif_	0.58 ± 0.14	0.73 ± 0.16	< 0.001^***^
CBF_max_ (mL/100 g/min)	56.90 (40.88–77.03)	105.45 (87.58–132.43)	< 0.001^***^
Volume (mL)	15.11 (8.26–33.23)	32.40 (15.76–47.84)	< 0.001^***^

Abbreviations: *PCNSL*, primary CNS lymphoma; *GBM*, glioblastoma; *rADC*_*max*_, relative maximum apparent diffusion coefficient; *rADC*_*mean*_, relative mean apparent diffusion coefficient; *rADC*_*min*_, relative minimum apparent diffusion coefficient; *rADC*_*dif*_, relative (max–min) apparent diffusion coefficient; *CBF*_*max*_, maximum cerebral blood flow

^*^p < 0.05

^**^p < 0.01

^***^p < 0.001

**Table 2:** Comparison of patient information, qualitative imaging features, and quantitative imaging parameters between atypical PCNSL, typical PCNSL, and GBM

	Atypical PCNSL (*n* = 29)	Typical PCNSL (*n* = 59)	GBM (*n* = 70)	*p* value
Age (years)	60.00 (45.50–66.50)	51.00 (45.00–62.00)	52.00 (43.00–58.00)	0.133
Sex				0.934
Male	17 (58.6%)	37 (62.7%)	43 (61.4%)	
Female	12 (41.4%)	22 (37.3%)	27 (38.6%)	
Location				0.172
Deep brain	19 (65.5%)	33 (55.9%)	32 (45.7%)	
Not deep brain	10 (34.5%)	26 (44.1%)	38 (54.3%)	
Number of lesions				0.236
Single	17 (58.6%)	32 (54.2%)	48 (68.6%)	
Multiple	12 (41.4%)	27 (45.8%)	22 (31.4%)	
rADC_max_	1.26 (1.17–1.41)	1.28 (1.18–1.35)	1.53 (1.39–1.59)	< 0.001 (1.000^*^, < 0.001^#^, < 0.001^&^)
rADC_mean_	0.94 ± 0.16	0.91 ± 0.12	1.18 ± 0.17	< 0.001 (1.000^*^, < 0.001^#^, < 0.001^&^)
rADC_min_	0.70 (0.55–0.79)	0.69 (0.59–0.78)	0.72 (0.66–0.84)	0.032 (1.000^*^, 0.097^#^, 0.086^&^)
rADC_dif_	0.61 ± 0.15	0.57 ± 0.13	0.73 ± 0.16	< 0.001 (0.849^*^, < 0.001^#^, < 0.001^&^)
CBF_max_ (mL/100 g/min)	53.20 (42.10–81.80)	58.00 (38.90–74.10)	105.45 (87.58–132.43)	< 0.001 (1.000^*^, < 0.001^#^, < 0.001^&^)
Volume (mL)	15.71 (6.95–33.71)	14.73 (9.54–33.58)	32.40 (15.76–47.84)	0.001 (0.849^*^, 0.002^#^, 0.036^&^)
nET volume ratio (× 100%)	0.15 (0.11–0.22)		0.31 (0.22–0.41)	< 0.001

^*^, #, & represent the* p* values compared by atypical PCNSL and typical PCNSL, atypical PCNSL and GBM, and typical PCNSL and GBM, respectively

Abbreviations: *PCNSL*, primary CNS lymphoma; *GBM*, glioblastoma; *rADC*_*max*_, relative maximum apparent diffusion coefficient; *rADC*_*mean*_, relative mean apparent diffusion coefficient; *rADC*_*min*_, relative minimum apparent diffusion coefficient; *rADC*_*dif*_, relative (max–min) apparent diffusion coefficient; *CBF*_*max*_, maximum cerebral blood flow; *nET*, non-enhancing tumor

### ADC, ASL-derived parameters, and tumor volume

**Fig. 4:**
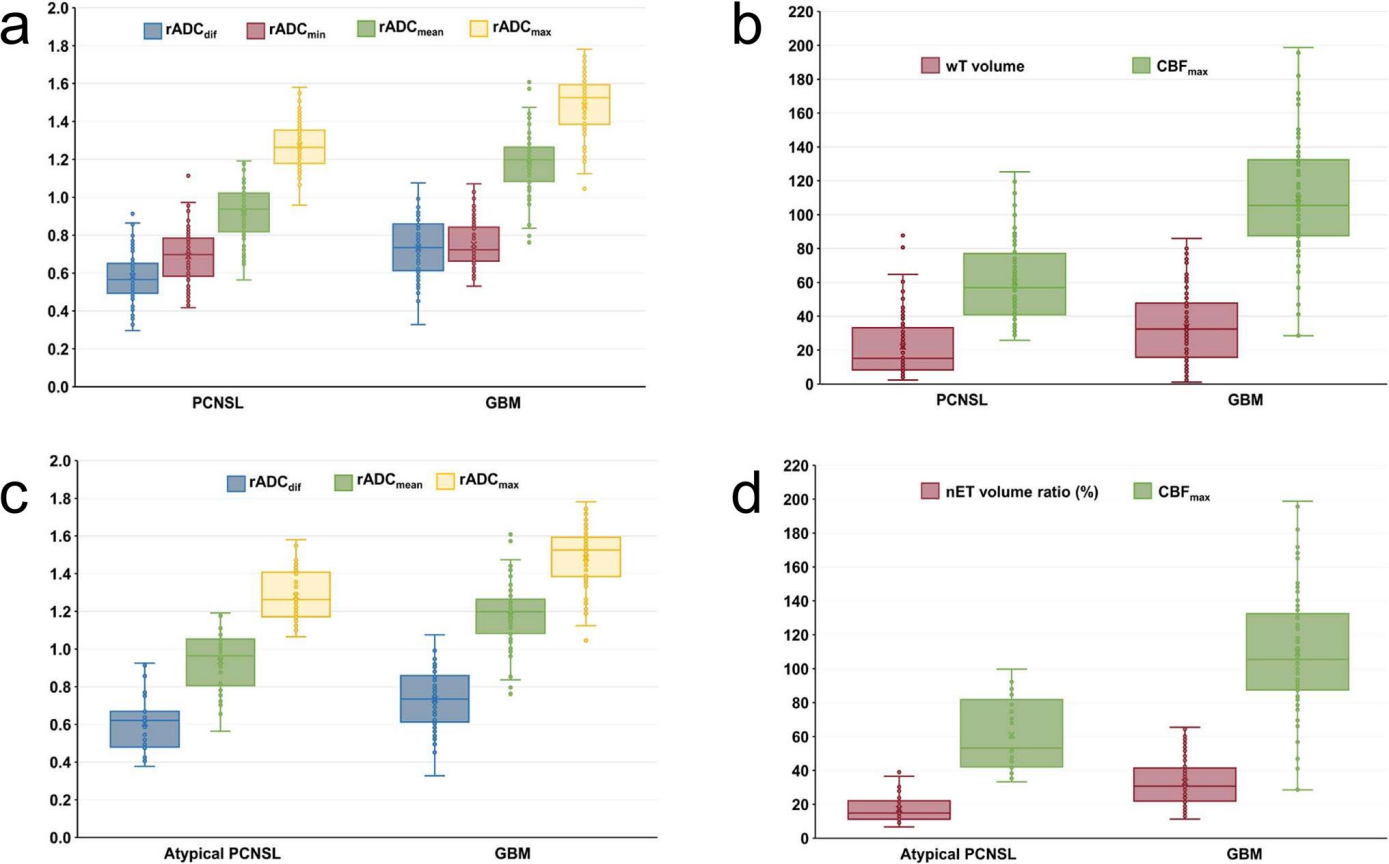
Box plots showing rADC_min_, rADC_max_, rADC_mean_, rADC_dif_, CBF_max_, volume, and nET volume ratio in patients with PCNSL and GBM and atypical PCNSL and GBM. **a** Boxplots of relative diffusion characteristics in patients with PCNSL and glioblastoma. **b **Boxplots showing volume and CBF_max_ in PCNSL and GBM. **c** Boxplots of relative diffusion characteristics in patients with atypical PCNSL and glioblastoma. **d** Boxplots showing CBF_max_ and nET volume ratio in atypical PCNSL and GBM. Boxes indicate interquartile range, lines in boxes indicate median values. The whiskers extend from the median to ± 1.5 × interquartile ranges. rADC_min_, relative minimum apparent diffusion coefficient; rADC_max_, relative maximum apparent diffusion coefficient; rADC_mean_, relative mean apparent diffusion coefficient; rADC_dif_, relative (max–min) apparent diffusion coefficient; CBF_max_, maximum cerebral blood flow; nET, non-enhancing tumor; PCNSL, primary CNS lymphoma; GBM, glioblastoma

**Fig. 5:**
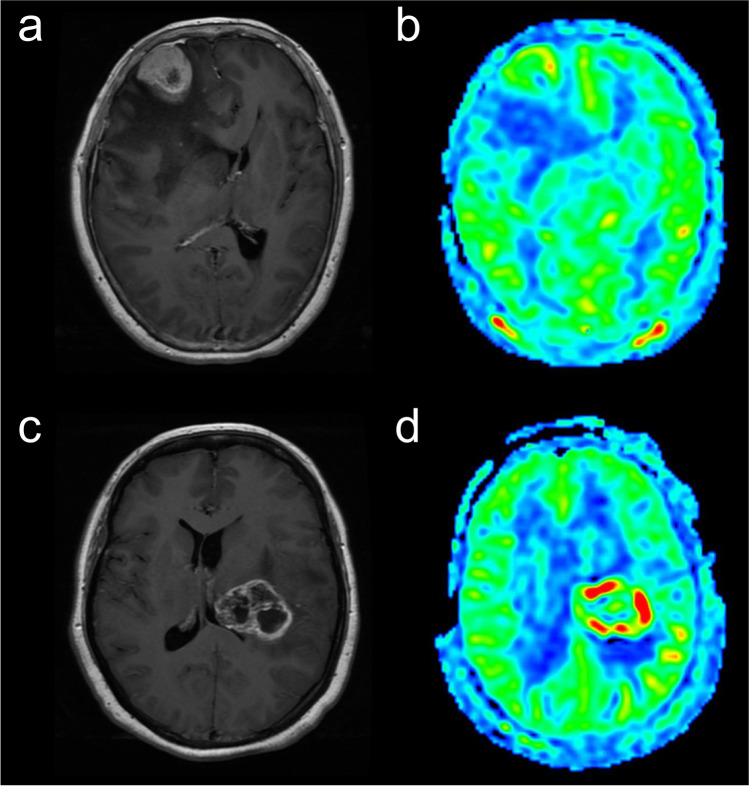
Representative enhanced T1WI and ASL perfusion map in patients with atypical PCNSL and GBM. **a** A 54-year-old female patient with PCNSL showing remarkable heterogeneous enhancement on a contrast-enhanced T1WI image. **b** Compared with the contralateral brain, the ASL perfusion map shows iso-perfusion in the tumor region. **c** A 52-year-old female patient with GBM showing irregular enhancement on a contrast-enhanced T1WI image. **d** Compared with the contralateral brain, the ASL perfusion map shows hyperperfusion in the tumor region. ASL, arterial spin labeling; PCNSL, primary CNS lymphoma; GBM, glioblastoma

The interobserver ICCs for ADC_max_, ADC_min_, ADC_mean_, ADC_NAWM_, CBF_max_, nET, and wT were 0.851, 0.982, 0.952, 0.971, 0.982, 0.982, and 0.988, respectively. The PCNSLs and GBMs quantitative imaging parameters are summarized in Table 1. The atypical PCNSLs, typical PCNSLs, and GBMs quantitative imaging parameters (ADC, ASL-derived, and tumor volume) are summarized in Table 2. The atypical PCNSLs and GBMs non-enhancing tumor volume ratio results are summarized in Table 2. Atypical PCNSLs and all PCNSLs showed significantly lower rADC_max_, rADC_mean_, and rADC_dif_ than that of GBMs (*p* < 0.05). GBMs showed significantly higher CBF_max_ and tumor volume than atypical PCNSLs and typical PCNSLs (*p* < 0.05). GBMs showed a significantly higher nET volume ratio than atypical PCNSLs (*p* < 0.05). The rADC_min_, rADC_max_, rADC_mean_, rADC_dif_, CBF_max_, volume, and nET volume ratio in patients with PCNSL and GBM and atypical PCNSL and GBM are shown in Fig. 4. The representative enhanced T1WI and ASL perfusion maps for atypical PCNSL and GBM are shown in Fig. 5.

### Single and multiple-parameter diagnostic performance

**Fig. 6:**
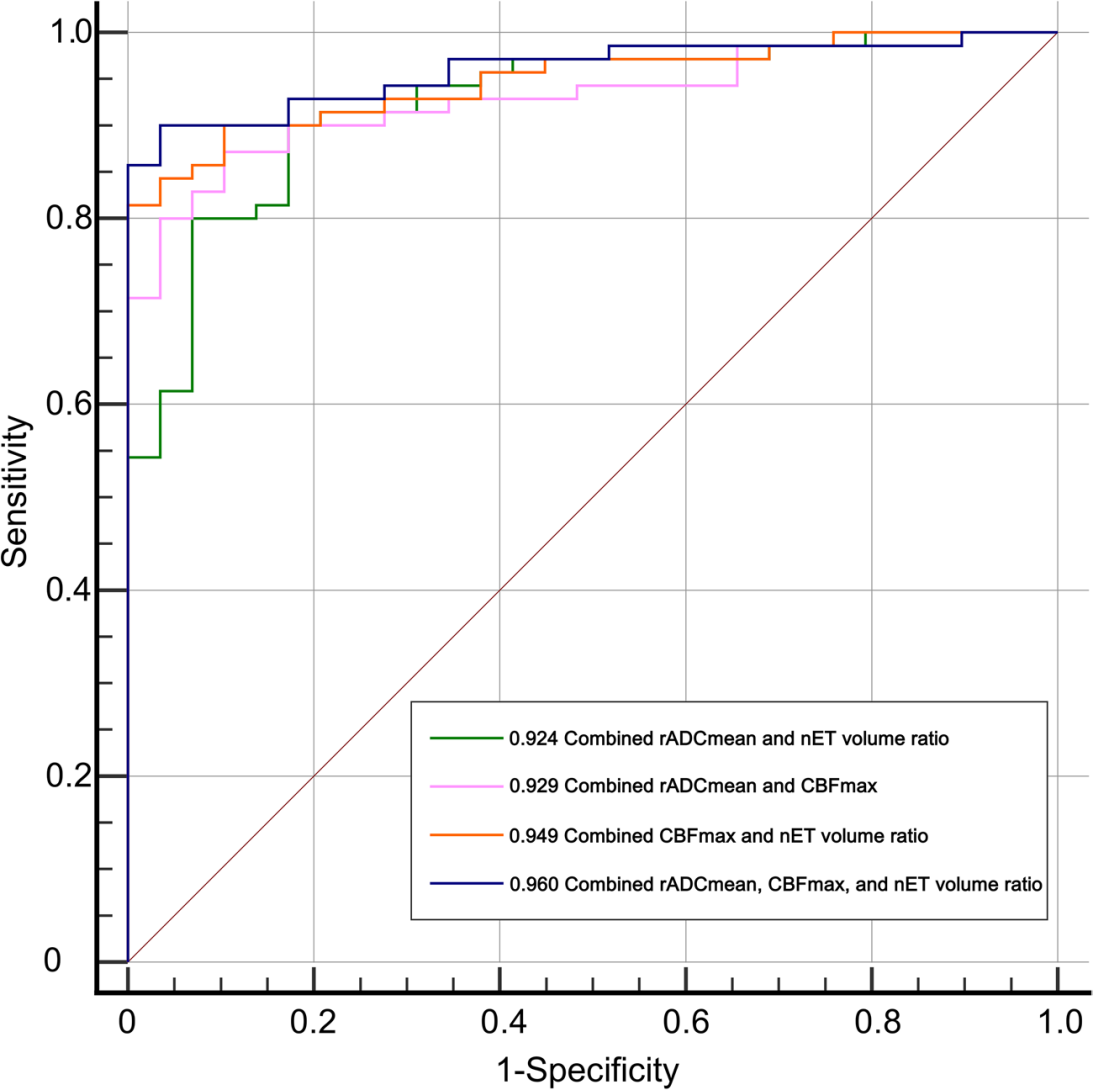
Receiver operating characteristic curves combining two and three parameters were compared to distinguish between atypical PCNSL and GBM. PCNSL, primary CNS lymphoma; GBM, glioblastoma; rADC_mean_, relative mean apparent diffusion coefficient; CBF_max_, maximum cerebral blood flow; nET, non-enhancing tumor

The single-parameter diagnostic performance results used to differentiate PCNSL and GBM are summarized in Table 3. The single-parameter diagnostic performance results used to differentiate atypical PCNSL and GBM are summarized in Table 4. The AUC of the rADC_mean_ was higher than rADC_max_ and rADC_dif_. The parameters rADC_mean_, CBF_max_, and nET volume ratio had the highest AUC value and were selected for assessment as a multiparameter. The multiparametric diagnostic performance results used to differentiate between atypical PCNSL and GBM are summarized in Table 5. The AUC of paired imaging parameters (rADC_mean_ and CBF_max_, rADC_mean_ and nET volume ratio, and CBF_max_ and nET volume ratio) was higher than that for any single parameter, and the three-variable combination (rADC_mean_, CBF_max_, and nET volume ratio) was superior to the two-variable combination. The AUC of the three-variable model (rADC_mean_, CBF_max_, and nET volume ratio) was 0.96, the sensitivity was 90%, and the specificity was 96.55%, which was superior to that of the two-variable model, as shown in Fig. 6.

**Table 3:** Diagnostic accuracy of ADC parameters, ASL-derived parameters, and volume for differentiating PCNSL from GBM

	AUC (95% CI)	Sensitivity (%)	Specificity (%)	Cut-off value
rADC_max_	0.847 (0.782, 0.900)	88.64	71.43	1.42
rADC_mean_	0.887 (0.827, 0.932)	90.91	78.57	1.06
rADC_min_	0.618 (0.538, 0.694)	26.14	95.71	0.58
rADC_dif_	0.766 (0.692, 0.829)	80.68	68.57	0.68
CBF_max_	0.881 (0.820, 0.927)	75.00	90.00	74.70
Volume	0.670 (0.591, 0.743)	59.09	74.29	16.51

Abbreviations: *CI*, confidence interval; *rADC*_*max*_, relative maximum apparent diffusion coefficient; *rADC*_*mean*_, relative mean apparent diffusion coefficient; *rADC*_*min*_, relative minimum apparent diffusion coefficient; *rADC*_*dif*_, relative (max–min) apparent diffusion coefficient; *CBF*_*max*_, maximum cerebral blood flow

**Table 4:** Diagnostic accuracy of ADC parameters, ASL-derived parameters, and non-enhancing volume ratio for differentiating atypical PCNSL from GBM

	AUC (95% CI)	Sensitivity (%)	Specificity (%)	Cut-off value
rADC_max_	0.829 (0.740, 0.897)	85.71	68.97	1.33
rADC_mean_	0.867 (0.785, 0.927)	74.29	93.10	1.11
rADC_dif_	0.727 (0.628, 0.812)	68.57	79.31	0.69
CBF_max_	0.882 (0.802, 0.938)	70.00	93.10	89.80
nET volume ratio	0.852 (0.766, 0.915)	70.00	86.21	0.25

Abbreviations: *CI*, confidence interval; *rADC*_*max*_, relative maximum apparent diffusion coefficient; *rADC*_*mean*_, relative mean apparent diffusion coefficient; *rADC*_*dif*_, relative (max–min) apparent diffusion coefficient; *CBF*_*max*_, maximum cerebral blood flow; *nET*, non-enhancing tumor

**Table 5:** Comparison of multiparameter models’ differentiation of atypical PCNSL and GBM

	AUC (95% CI)	Sensitivity (%)	Specificity (%)
rADC_mean_ and CBF_max_	0.929 (0.859, 0.971)	87.14	89.66
rADC_mean_ and nET volume ratio	0.924 (0.853, 0.967)	80.00	93.10
CBF_max_ and nET volume ratio	0.949 (0.885, 0.983)	81.43	100.00
rADC_mean_, CBF_max_, and nET volume ratio	0.960 (0.900, 0.989)	90.00	96.55

Abbreviations: *PCNSL*, primary CNS lymphoma; *GBM*, glioblastoma; *AUC*, area under curve; *CI*, confidence interval; *rADC*_*mean*_, relative mean apparent diffusion coefficient; *CBF*_*max*_, maximum cerebral blood flow; *nET*, non-enhancing tumor

## Discussion

This study first compared patient information, qualitative imaging features, and quantitative imaging parameters of all PCNSLs (including 29 atypical PCNSLs and 59 typical PCNSLs) and GBMs. Then, the patient information, qualitative imaging features, and quantitative imaging parameters of atypical PCNSLs, typical PCNSLs, and GBMs were compared. Among the 29 patients with atypical PCNSL, 18 (62.1%) underwent subtotal or total resection because of preoperative misdiagnosis and significant space-occupying effect. To reduce the incidence of preoperative misdiagnosis as much as possible, we evaluated multiparameter diagnostic performance using ADC, ASL, and nET volume ratio to distinguish between atypical PCNSL and GBM in this study. The three-variable (rADC_mean_, CBF_max_, and nET volume ratio) model has an AUC of 0.96, a sensitivity of 90%, and a specificity of 96.55% and can reliably distinguish atypical PCNSL from GBM, having a higher AUC than any single and two-variable evaluation.

Given that all 158 cases (70 GBMs, 29 atypical PCNSLs, and 59 typical PCNSLs) showed obvious enhancement, enhancing and non-enhancing volumes were regarded as the tumor volume. Previous studies reported [8] that there was no significant difference in tumor volume between PCNSL and GBM. Our study showed that the tumor volume of both atypical PCNSL and all PCNSL groups is significantly smaller than that of the GBM group, which is consistent with previous studies [19]. We found that, compared with GBM patients, atypical PCNSL patients showed a significantly lower nET volume ratio. Choi et al [11] used the semiautomatic signal intensity threshold to calculate an optimal cut-off necrosis ratio of 13% for differentiating PCNSL and GBM. A recent study [22] used the RadioFusionOmics (RFO) model to differentiate between GBM and solitary brain metastasis, which included the volume of the non-enhancing and enhancing tumor and peritumoral edema. In this study, we attempted to differentiate atypical PCNSL patients from GBM patients using nET volume quantification. Youden’s index showed that an optimal cut-off nET volume ratio of 25% can be used to distinguish between atypical PCNSL and GBM. Although the study included atypical PCNSL (hematology, necrosis, or heterogeneous contrast enhancement), the nET volume ratio of the atypical PCNSL (14.9%) was significantly lower than that of GBM (30.8%). The possible reason is that GBMs are highly heterogeneous at molecular and histological levels with the tissue, including extensive pseudopalisading necrosis and microvascular proliferation [23].

Although ADC values are independent of hardware and field strength under fixed parameters [24], to consider possible interindividual variations in brain diffusivity, this study standardized various ADC values, providing further advantages for brain MRI performed at different scanners. ROIs are placed in the solid component of the tumor to minimize the impact of the partial-volume effect on the results, and our study showed that interobserver reproducibility was good to excellent for ROI measurement ADC (intraclass correlation coefficient, 0.85–0.98). A previous study [6] showed that the AUC of ADC_min_ to distinguish atypical PCNSL and GBM was 0.71–0.73. In this study, rADC_min_, rADC_max_, rADC_mean_, and rADC_dif_ better reflected the diffusion heterogeneity tumor cellularity. Our results showed that the rADC_max_, rADC_mean,_ and rADC_dif_ of atypical PCNSL and all PCNSL were lower than of GBM, which is consistent with previous results [12, 14, 16, 18], and rADC_mean_ showed an optimal diagnostic efficiency in differentiating atypical PCNSL from GBM (AUC = 0.867). Furthermore, it indicates that the lower ADC in patients with atypical PCNSL may be related to the more limited diffusion caused by higher tumor cell density compared with patients with GBM [25, 26]. Our results show that the combination of multiple parameters (AUC = 0.96) can improve diagnostic efficiency. A previous study using diffusion radiomics to distinguish between atypical PCNSL and GBM had an AUC of 0.984 [19], slightly higher than with our combined multiparameter (AUC = 0.96). However, our study, which included the quantification of diffusion, perfusion, and volume of non-enhanced areas, will have better clinical applicability and feasibility to differentiate between atypical PCNSL and GBM.

ASL-MRI requires no external tracer perfusion and has a demonstrated ability to differentiate GBM from PCNSL [10, 15, 27]. Since previous studies did not compare the ASL parameters of typical PCNSL and atypical PCNSL, we first compared the CBF_max_ of typical lymphoma and atypical PCNSL. The results showed that the difference was not statistically significant. We subsequently compared the ASL parameters of atypical PCNSL and GBM in this study. The present results showed that the CBF_max_ of atypical PCNSLs was significantly lower than that of GBMs, which was consistent with previous studies [10, 15, 27]. The AUC of CBF_max_ differentiating atypical PCNSL from GBM is 0.882, and the cut-off value is 89.8 mL/100 g/min. The reason for this result is that the extensive neovascularization of GBM leads to hyperperfusion [7, 23, 28], a condition that is absent in PCNSL [29, 30].

Our study has some limitations. First, this was a relatively small retrospective study due to the strict inclusion criteria and low incidence of PCNSL; therefore, larger studies are required to verify our results. Second, due to the extremely irregular shape of GBM, when measuring the ROI of ADC placement, we selected the area with more tumor solid components (multiple random larger layers) for measurement, which may have affected the results to some extent. Lastly, for the delineation of the volume of non-enhanced areas, especially for patients with GBM, there are mixed enhanced and minuscule non-enhancing areas in the tumor, making it difficult to delineate ROIs. This may have affected the results to some extent.

In conclusion, multiple parameters (non-enhancing volume, ADC, and ASL) perform well in distinguishing atypical PCNSL and GBM and have good clinical feasibility and practicability.

## Supplementary Information

Below is the link to the electronic supplementary material.

Supplementary file1 (ZIP 126 kb)

## Abbreviations

3D-ROI: Three-dimensional region of interest
ADC_NAWM_: Normal appearing white matter apparent diffusion coefficient
AIDS: Acquired immune deficiency syndrome
ASL: Arterial spin labeling
CBF: Cerebral blood flow
GBM: Glioblastoma
nET: Non-enhancing tumor
PCNSL: Primary central nervous system lymphoma
rADC_dif_: Relative (max–min) apparent diffusion coefficient
rADC_max_: Relative maximum apparent diffusion coefficient
rADC_mean_: Relative mean apparent diffusion coefficient
rADC_min_: Relative minimum apparent diffusion coefficient
VOI: Volume of interest
wT: Whole tumor

## Declarations

### Guarantor

The scientific guarantor of this publication is Liangping Luo.

### Statistics and biometry

No complex statistical methods were necessary for this paper.

### Methodology

• retrospective

• cross-sectional study

• performed at one institution
