# Supplementary material for: Atypical primary central nervous system lymphoma and glioblastoma: multiparametric differentiation based on non-enhancing volume, apparent diffusion coefficient, and arterial spin labeling
Source: Eur Radiol. 2023 May 12;33(8):5357–67. doi: 10.1007/s00330-023-09681-2 (PMC10326108; doi:10.1007/s00330-023-09681-2)
Supplement: Supplementary file 1 — Supplementary file1 (ZIP 126 kb) [file 330_2023_9681_MOESM1_ESM.zip › EURA-D-22-02078_ESM/EURA-D-22-02078_ESM_Table S1.pdf]

ELECTRONIC SUPPLEMENTARY MATERIAL

Atypical Primary Central Nervous System Lymphoma and Glioblastoma: Multiparametric Differentiation based on Non-enhancing Volume, Apparent Diffusion Coefficient, and Arterial Spin Labeling

Supplementary Table 1 Operation modes and intraoperative pathological diagnosis of typical and atypical PCNSL.

	**Atypical PCNSL (n = 29)**	**Typical PCNSL (n = 59)**
Operation mode		
Stereotactic biopsy	11 (37.9%)	42 (71.2%)
Subtotal or total resection	18 (62.1%)	17 (28.8%)
Intraoperative pathological consultation	24 (82.68%)	54 (91.5%)
Intraoperative pathological diagnosis		
Lymphoma	22 (91.7%)	46 (85.2%)
Round cell malignant tumor	2 (8.3%)	7 (13.0%)
High-grade glioma	0 (0.0%)	1 (1.8%)

Abbreviations: PCNSL, primary CNS lymphoma
